# PET Visualized Stimulation of the Vestibular Organ in Menière's Disease

**DOI:** 10.3389/fneur.2020.00011

**Published:** 2020-01-28

**Authors:** Louise Devantier, Allan K. Hansen, Jens-Jacob Mølby-Henriksen, Christian Bech Christensen, Tina Lildal, Michael Pedersen, Måns Magnusson, Per Borghammer, Therese Ovesen

**Affiliations:** ^1^Department of Clinical Medicine, Aarhus University, Aarhus, Denmark; ^2^Department of Oto-Rhino-Laryngology, Regional Hospital West Jutland, Holsterbo, Denmark; ^3^Department of Nuclear Medicine & PET Centre, Aarhus University Hospital, Aarhus, Denmark; ^4^Department of Engineering, Aarhus University, Aarhus, Denmark; ^5^Comparative Medicine Lab, Aarhus University, Aarhus, Denmark; ^6^Department of Oto-Rhino-Laryngology, Lund University Hospital, Lund, Sweden

**Keywords:** Menière's disease, central vestibular system, vestibular cortex, positron emission tomography, neuroimaging

## Abstract

**Introduction:** The cortical metabolic activity in patients with Menière's disease has not been investigated. The aim of this study was to investigate the ^18^F-FDG cerebral uptake in Menière's patients compared to healthy controls.

**Method:** Eight patients with right-sided Menière's disease and fourteen healthy controls underwent a video head impulse test (vHIT), test of utricular function with ocular vestibular evoked myogenic potentials (oVEMP) and three ^18^F-FDG-based PET examinations of the brain. Participants were seated in a self-propelled chair, injected with ^18^F-FDG and then exposed to 35 min of chair motion stimulation, followed by a PET scan. Two types of natural vestibular stimuli were applied, predominantly toward the right horizontal semicircular canal (angular acceleration) and right utriculus (linear acceleration). For baseline scans, participants were injected with ^18^F-FDG while seated without movement.

**Results:** Analyses of baseline scans revealed decreased ^18^F-FDG-uptake in the medial part of Heschl's gyrus in the left hemisphere in patients with Menière's disease compared to healthy controls. During angular vestibular stimulation there was also a significantly decreased ^18^F-FDG uptake in the intersection between the medial part of Heschl's gyrus and the parietal operculum in the left hemisphere and bilaterally in the posterior part of insula. During linear stimulation, Menière's patients showed decreased ^18^F-FDG uptake in the medial part of Heschl's gyrus in the right hemisphere and also bilaterally in the posterior insula. In addition, decreased ^18^F-FDG uptake was seen in the thalamus during vestibular stimulation.

**Conclusion:** Heschl's gyrus, the posterior part of insula, and thalamus have previously been shown to be core areas for processing vestibular inputs. Patients with Menière's disease solely differed from the healthy controls with lower cortical activity in these areas at baseline and during natural vestibular stimulation.

## Introduction

Menière's disease is characterized by spontaneous episodes of vertigo combined with tinnitus, aural fullness, and fluctuating low frequency sensorineural hearing loss. The diagnosis of Menière's disease is solely dependent on symptoms and exclusion of other neurological and neuro-otological diseases (1). The etiology of Menière's disease is uncertain (2). Endolymphatic hydrops is considered a hallmark even though it is unable to account for all the clinical features of this disease (3). Thus, it is important to investigate how Menière's disease alters neurological processing of vestibular stimuli and relate this relationship to the clinical features of this disease. The location of a distinct vestibular cortex in humans has long been debated (4). We recently provided evidence that the primary vestibular cortex may be located in the most medial part of Heschl's gyrus (5). Those results were obtained from ^18^F-FDG-PET brain scans, showing a cortical response mainly in Heschl's gyrus during 35 min of natural vestibular stimulation.

In the early stages of the Menière's disease, the vestibular function is rarely affected between vertigo spells. However, hearing loss and loss of vestibular function seem to gradually evolve over time despite various treatment modalities (6). Loss of sensory afferents leads to multisensory compensation in the brain, which is also believed to account for the clinical recovery in patients with vestibular disorders (7). Neuroimaging of patients with vestibular loss has shown functional cortical changes within the visual and proprioceptive areas compared to healthy controls (7–10). Hitherto, it is unknown if the central processing of vestibular stimuli in patients with Menière's disease differs from that in healthy subjects or patients with other peripheral vestibular diseases.

Thus, the aim of this study was to investigate the ^18^F-FDG cerebral uptake in Menière's patients compared to healthy controls.

## Materials and Methods

### Participants With Menière's Disease

A total of eight patients with Menière's disease (five females; age: 50–62 years, median 57 years) were recruited. All had definitive Menière's disease on their right ear according to the Bárány Society diagnostic criteria (1). Two participants had vestibular migraine in conjunction with Menière's disease. Disease duration ranged from 2 to 9 years. Only right-handed patients according to the 10-item inventory of the Edinburg test (11) and non-smokers were included. All patients were interviewed to exclude other neurological disorders, psychiatric disorder, and use of potentially neuromodulating medication. One patient received no treatment, three were treated with tympanostomy in the tympanic membrane (a grommet), one with betahistine (8 mg × 1) and one with a combination of a grommet and betahistine (8 mg × 3). Two patients were treated with a combination of the Meniett device and betahistine (16–32 mg × 3). Vestibular function was evaluated by cervical vestibular evoked myogenic potentials (cVEMP), air-conducted Hz short tone bursts 500 Hz (Eclipse, Interacoustics, Denmark), ocular vestibular evoked myogenic potentials (oVEMP), bone-conducted Hz short tone bursts 500 Hz (Eclipse, Interacoustics, Denmark), (12) and video head impulse test (vHIT) (EyeSeeCam, Interacoustics, Denmark) (13). In order to validate the diagnosis of definitive Menière's disease all participants provided a full audiometry showing a unilateral low- to mid-frequency sensorineural hearing loss on their right ear. One patient had normal hearing between attacks. In addition to a low- to mid- frequency sensorineural hearing loss, two patients also had high-frequency hearing loss (Table 1). One of the patients with high frequency hearing loss also suffered from presbyacusis on the left ear. The rest of the participants had normal hearing on the left ear.

**Table 1:** MD patients.

**Frequency (Hz)**	**250**	**500**	**1,000**	**2,000**	**4,000**	**8,000**
Threshold (dB nHL)	60 (20–70)	62 (20–75)	60 (20–85)	45 (20–90)	35 (15–80)	40 (20–80)

*Hearing loss in the right ear*.

*MD patients all documented sensorineural hearing loos in the right ear with an audiogram. One patient had normal hearing between attacks. Pure tone thresholds, median (range) are shown for the different frequencies*.

### Healthy Participants

The healthy control group included 14 healthy participants (eight females; age: 50–60 years, median 55 years). Similar to the Menière's patients, they were all right-handed, non-smokers, had no neurological, or psychiatric disorders and used no neuromodulating medication.

Informed written and oral consent were obtained from all participants. Central Region of Denmark Research Ethics Committee approved the study (no: 1-10-72-135-16).

### ^18^F-FDG PET Procedures

**Figure 1:**
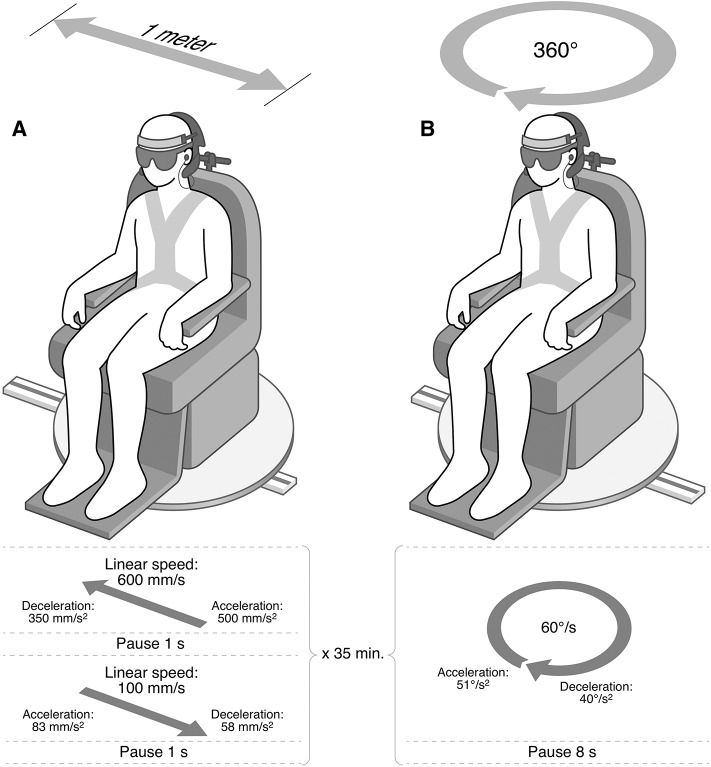
The self-propelled chair. The participants were seated in the self-propelled chair. During vestibular stimulation and baseline they wore noise-canceling earphones and sleep-goggles. **Angular stimulation**: The chair rotated 360° clockwise (Acceleration: 51°/s^2^, speed: 60°/s) followed by an 8 s pause before initiating the next rotation. The chair repeated this pattern for 35 min. The chair performed clockwise rotations in order to create a predominantly right-sided stimulus of the semicircular canal. **Linear stimulation**: The chair moved rapidly to the right (acceleration: 500 mm/s^2^, speed: 600 mm/s) then paused for 1 s before slowly (Acceleration: 83 mm/s^2^, speed: 100 mm/s) returning to the starting position. This movement pattern was repeated for 35 min. The chair performed cycles of rapid rightward movements and slow leftward movements during 35 min of stimulation in order to create a predominantly right-sided utricular stimulus.

Three ^18^F-FDG PET scans were performed on 3 individual days; after angular and linear stimulation of the vestibular organ, respectively, in addition to one baseline ^18^F-PET scan. None of the patients had a Menière's attack in the week prior to the ^18^F-FDG PET scans. Before each ^18^F-FDG PET examination, the patient was tested for spontaneous nystagmus with video-nystagmography (VNG) goggles (VO425, Interacoutics, Middelfart, Denmark), and a head shake-test was performed. All participants had fasted 6 h prior to the bolus injection of radioactive ^18^F-FDG [170 MBq (±10%)]. Natural vestibular stimulation was carried out in a custom-designed self-propelled chair (Figure 1). All subjects wore noise-canceling in-ear headphones (QuietControl-30 wireless headphones, Bose, USA) and sleep-goggles to minimize auditory input avoid and visual stimulation. The participant's head was fixated in a headgear ~20° downward. Immediately after the bolus injections, the self-propelled chair was set in motion or it was motionless (baseline scan) for 35 min prior to the ^18^F-FDG PET scan.

### Vestibular Stimulation and Baseline

The participant was immobilized in a face-forward position during the 35 min of stimulation in the chair. A schematic illustration of the chair and the movement patterns of the chair are shown in Figure 1. The order of the stimulation paradigms was randomized.

#### Linear Stimulation

The chair moved to the right (acceleration: 500 mm/s^2^, speed: 600 mm/s), and then paused for 1 s before slowly (acceleration: 83 mm/s^2^, speed: 100 mm/s) returning to the starting position. This movement pattern was repeated for 35 min.

#### Angular Stimulation

The chair rotated 360° clockwise (Acceleration: 51°/s^2^, speed: 60°/s) then paused for 8 s before initiating the next rotation. The chair repeated this pattern for 35 min (Figure 1).

#### Baseline Condition

The chair was kept motionless for 35 min.

### PET Acquisition

After the stimulation, the participants were transferred to a wheel chair and transported to the PET facility, while still wearing noise-cancellation earphones and sleep-goggles. Each participant was placed comfortably on the bed of the high-resolution PET system (ECAT HRRT; CTI/Siemens, Knoxville, TN, USA). The PET scan protocol has previously been reported (14). At 54 min post-injection, a 6 min transmission scan (Cs-137 point source) was performed. At exactly 60 min post-injection, the 60 min dynamic PET acquisition (12 × 5 min frames) was initiated. An ordered-subsets expectation maximization 3D algorithm was used to reconstruct the PET scans into image volumes consisting of 207 axial slices and a 1.22 mm voxel size. Frame-to-frame motion correction was made followed by summation of all frames into one static PET data volume. Reconstructed images were revised to detect dead time, random, and scatter events, and detector efficiency variations.

### MRI Acquisition

MRI was performed with a 3 T system (Siemens Skyra, Siemens Healthcare, Germany) using a 32-element head-coil for data reception. Two MRI sequences were acquired. First, a T2-weighted spin echo sequence was employed in the axial direction for high-resolution imaging of the inner ear, with these parameters: TE = 152 ms, TR = 4,540 ms, image resolution 0.9 × 0.9 × 0.9 mm^3^, acquisition time = 5.5 min. Second, a full brain 3D T1-weighted gradient-echo sequence was performed using the parameters: TE = 1.5 ms, TR = 16 ms, flip angle = 15°, 0.9 × 0.9 × 0.9 mm^3^, acquisition time = 5 min.

### PET Data Analysis

The three PET image volumes from each participant were co-registered to common stereotactic space (MNI space) via each individual's MRI using SPM12 (15) for MATLAB (MathWorks, Natick, Massachusetts, USA). Automated gray matter-white matter segmentation was performed on the T1-weighted MRI images. To minimize effects of irrelevant global scaling factors in the FDG activity levels, the FDG values were intensity-normalized (using ratio-normalization) to the mean activity level of the total gray matter by applying a gray matter volume of interest (VOI), thus obtaining standard uptake value ratio (SUVR) maps. Two VOIs defining the bilateral posterior insula and the bilateral Heschl's gyrus were obtained using SPM's built-in atlas. Averaged SUVR values from these VOIs were obtained to test the *a priori* hypothesis that these VOIs cover primary cortical areas for processing information from the vestibular organ. To compare the baseline brain activity between the two groups, baseline maps from patients with Menière's disease were contrasted with the baseline maps from the healthy controls Stimulus-induced activations were interrogated by creating ΔSUVR images of each type of stimulation compared to the baseline SUVR, e.g., ΔSUVR_difference_ = SUVR_stimulation_-SUVR_baseline_. These ΔSUVR_difference_ images were then compared between Menière patients and controls using simple two-tailed Student's *t*-test. A separate analysis was performed for the linear and angular paradigm, respectively, Correlation between activation and oVEMP was interrogated using a linear regression model with the lateralized (left/right) oVEMP response result (positive/negative) as independent variables and mean VOI ΔSUVR_difference, linear_ as the dependent variable. *P* < 0.05 were considered statistically significant. SUVR maps were smoothed using a 12 mm Gaussian filter, before a voxel-based statistical analysis of the FDG ΔSUVR_difference_ images of each type of vestibular stimulation was performed within gray matter voxels using simple voxel-wise *t*-tests. Multiple comparison correction was performed using the built-in family wise error (FWE) correction with a threshold of *p* < 0.05.

## Results

Five patients showed no oVEMP response on the right (sick) side. The oVEMP response was absent bilaterally in three of these patients. Three patients had a normal oVEMP response. All had normal horizontal vHIT examination. All healthy participants had normal vestibular function evaluated by cVEMP, oVEMP, and vHIT.

The presented ^18^F-FDG PET scan results from patients with Menière's disease comprise eight baseline ^18^F-FDG PET scans, seven ^18^F-FDG PET scans after rotatory vestibular stimulation and seven ^18^F-FDG PET scans after linear vestibular stimulation. One patient suffered a classic Menière's attack during one of the PET scan visits and one PET scan failed. All 14 healthy controls underwent all three ^18^F-FDG PET scans.

### VOI-Based Analysis

Baseline ^18^F-FDG-uptake revealed a statistically significant lower activation in the right Heschl's gyrus (*p* = 0.002) in Menière's patients compared to healthy controls. There was no statistically significant difference found in baseline ^18^F-FDG-uptake in the bilateral posterior insula between the patients and healthy controls (*p* = 0.41) or in the left Heschl's gyrus (*p* = 0.19). During linear vestibular stimulation significantly lower ^18^F-FDG-uptake was seen in both the right Heschl's gyrus (*p* = 0.004) and the left Heschl's gyrus (*p* = 0.026) (Table 2). During angular stimulation there was also a significantly lower ^18^F-FDG-uptake in the right Heschl's gyrus (*p* = 0.005) but not on the left side (*p* = 0.16).

**Table 2:** Comparison of ^18^F-FDG uptake.

**Anatomical location**	**Bilat. Post. insula**	**Left Heschl's gyrus**	**Right Heschl's gyrus**
^18^F-FDG uptake			
Baseline comparison	0.415	0.193	0.002*
Angular stimulation	0.031*	0.156	0.005*
Linear stimulation	0.093	0.026	0.004*
Δ (Angular—baseline)	0.020*	0.914	0.371
Δ (Linear—baseline)	0.009*	0.219	0.182

Comparison of ^18^F-FDG uptake as mean SUVR in the predefined VOI between healthy participants and patients with Menierèr's disease. Statistically significant values are bold and marked with *.

In the comparison of delta-^18^F-FDG-uptake during vestibular stimulations with the baseline subtracted (ΔSUVR_difference_), significantly reduced ^18^F-FDG-uptake was observed during both linear (*p* = 0.009) and angular (*p* = 0.02) stimulation in the bilateral posterior insula. No differences in activation were as observed in Heschl's gyrus. Of note, the attenuated activation in posterior insula did not seem to be driven by group differences at baseline (*p* = 0.4), but by diminished increase of FDG uptake during stimulation (linear *p* = 0.093, angular *p* = 0.031) (Table 2).

There was no statistically significant difference between the group of five patients with absent oVEMP response and the three patients with normal oVEMP response. Activation in the posterior insula and Heschl's gyrus during the linear vestibular stimulation did not correlate with vestibular function measured by oVEMP response.

### Voxel-Based Analysis

**Figure 2:**
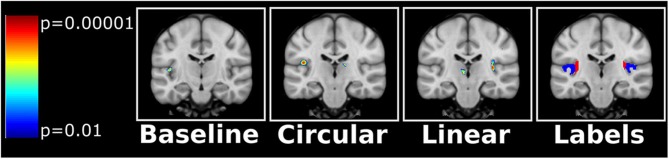
Uncorrected t-maps from the SPM analyses. Decreased ^18^F-FDG uptake was evident in Heschl's gyrus in the patients with Menière's disease compared to the healthy control group. During linear vestibular stimulation the Menière's patient displayed a decreased ^18^F-FDG uptake in the right Heschl's gyrus and a cluster in the left thalamus. However, during angular stimulation (circular) the Menière's patient displayed a decreased ^18^F-FDG uptake in the left Heschl's gyrus/intersection to the parietal operculum. The anatomical regions as defined in the SPM atlas are shown on the right (blue label = Heschl's gyrus, red label = posterior insula). Orientation by neurological convention: Left side of image is left side of brain.

No clusters survived FWE-correction, but some trends were observed. In baseline ^18^F-FDG PET scans, reduced cerebral ^18^F-FDG uptake was found in Menière's patients localized to the deep part of the left Heschl's gyrus compared to the healthy individuals. During the predominantly right-sided linear vestibular stimulation decreased cerebral ^18^F-FDG uptake was found in the deep part of Heschl's gyrus in the right hemisphere and in thalamus in the left hemisphere. During the clockwise angular vestibular stimulation a decreased cerebral ^18^F-FDG uptake was found in intersection between the medial part of Heschl's gyrus and parietal operculum on the left side (Figure 2).

## Discussion

Patients with Menière's disease showed significantly lower cerebral uptake of ^18^F-FDG in the most medial part of Heschl's gyrus in the left hemisphere during baseline ^18^F-FDG PET scan compared to healthy control. During the linear stimulation the patients also showed lower ^18^F-FDG uptake in the most medial part of Heschl's gyrus and the posterior insula in the right hemisphere. During angular stimulation a decreased cerebral ^18^F-FDG uptake was found on the left side in the intersection between the medial part of Heschl's gyrus and parietal operculum.

A primary vestibular cortex and a secondary association area has previously been hypothesized to be located in the most medial part of Heschl's gyrus and the posterior part of the insula (5). This location seems plausible given that the cochlea and vestibular part of the labyrinth share many familiarities. It is interesting that the voxel-based analysis comparison of baseline ^18^F-FDG PET scans only displayed a difference between the two groups in one area, namely decreased uptake in the medial part of Heschl's gyrus in patients with Menière's disease. Heschl's gyrus is perceived to be the primary auditory cortex, so it must be considered, whether the decreased ^18^F-FDG uptake in this particular area could be confounded by decreased auditory stimulus in the patients with Menière's disease. The patients with Menière's disease had a low- to mid-frequency hearing loss on the right ear and normal hearing on the left ear, with the exception of one patient with presbyacusis. Consequently, if the decreased ^18^F-FDG uptake was due to hearing loss we would have expected it to be located in the more lateral part of Heschl's gyrus given the tonotopic map of Heschl's gyrus (5). However, little is known about the interaction between the auditory and vestibular systems (16).

In the voxel-based analysis comparison of baseline ^18^F-FDG PET scans between the two groups revealed significant lower activity in the left hemisphere in the medial part of Heschl's gyrus and posterior insula (Figure 2). This finding was surprising given that it is contralateral to the Menière's ear. Vestibular inputs are believed to be mediated bilaterally but predominantly to the non-dominant hemisphere (17). However, lateralization of sensory inputs has been suggested to be context-dependent (18).

In 2003, Dieterich et al. reported dominance of vestibular cortical function in the non-dominant hemisphere in a H_2_O PET study during caloric stimulation (17). We assume that is impossible to confine a natural vestibular stimulation to one particular part of the vestibular organ during a time span of 35 min, so the goal of the linear vestibular stimulation was to create a primarily right-sided utricular stimulus in right-handed participants. oVEMP measurements are believed to reflect utricular function (19, 20). Five out of our eight patients had absent oVEMP responses on the right side. During the linear vestibular stimulation, Menière's patients showed significantly lower ^18^F-FDG uptake in the most medial part of Heschl's gyrus and the posterior insula in the right hemisphere. This finding was clearly demonstrated in the predefined VOI-based analysis and supported by the voxel-based analyses. Interestingly, this particular cortical area was the only area displaying a difference in ^18^F-FDG PET uptake between the two groups. The angular vestibular rotation was designed to primarily stimulate the right horizontal semicircular canal. However, the acceleration and deceleration during angular stimulation might not have differed significantly to create a primarily right-sided stimulus. All participants, healthy and controls, had normal vHIT results, indicating normal functioning horizontal semicircular canals. However, patients with Menière's disease had a decreased cerebral ^18^F-FDG PET uptake located in the intersection between the medial part of Heschl's gyrus and the parietal operculum and in the posterior part of insula compared to the control group.

Central multisensory compensation is believed to play a crucial role in patients with loss of vestibular function. To the best of our abilities, we designed the study to deprive the participants of visual, auditory, and proprioceptive inputs during baseline and vestibular stimulation. This approach was made in the attempt to create an uncorrupted stimulus to the vestibular organ by minimizing confounding co-activations of other sensory systems. This study design may explain why no increases in ^18^F-FDG-uptake were seen in other cortical areas. Another intriguing finding seen during linear vestibular stimulation was a reduction of ^18^F-FDG PET uptake in the left thalamus. The thalamus is generally considered a relay station that modulates sensory information flow to the cortex (21). Previous studies in both humans and animals reported findings suggestive of a vestibular-thalamic pathway (18, 22).

### Limitations

The sample size was limited to eight patients with Menière's disease, and only seven ^18^F-FDG PET scans during linear vestibular stimulation due to an unforeseen Menière attack in one patient and seven due to a failed PET scan. Thus, it is crucial that the present findings be replicated in an independent group of Meniere's patients. Fear of provoking a Menière's attack during vestibular stimulation was a concern for several participants. The inclusion and exclusion criteria were fairly strict in terms of age, disease duration, handedness, and previous medical history. However, there was no restriction with regards to vestibular function. Additional tests of the Menière's patients as for instance VEMP at different frequencies (23) or MRI with gadolinium would have been preferable (24). It is possible that the reduction in ^18^F-FDG uptake is more dependent on vestibular function than the etiology of the vestibular loss. In the VOI analysis the entire Heschl's gyrus was used in the analysis well-knowing that it mainly encompasses the primary auditory cortex. Thus, it is possible that the reported between-group difference in Heschl's gyrus was attenuated by the application of these large VOIs.

## Summary

Patients with Menière's disease displayed lower cortical activity in the intersection between Heschl's gyrus and posterior insula at baseline and during vestibular stimulation. All patients had Menière's disease in their right ear and interestingly the decreased ^18^F-FDG uptake during baseline was seen in the left hemisphere. During the primarily right-sided linear vestibular stimulation the decrease was seen in the same anatomical region but in the right hemisphere. All patients and healthy controls had normal function of their horizontal semicircular canal measured by vHIT, but a decreased cerebral ^18^F-FDG uptake was detected during angular vestibular stimulation in the left intersection between Heschl's gyrus and parietal operculum. No other cortical differences were seen, which would have been suggestive of central multisensory compensation. However, that particular finding may be due our study design, in which other sensory inputs were deliberately minimized.

This study it the first to visualize and compare physiological vestibular stimulation in a group of healthy participants and a group with Menière's disease. Patients with Menière's disease differed from healthy participants with a decreased ^18^F-FDG uptake during baseline and vestibular stimulation in the medial part of Heschl's gyrus, the posterior part of insula and thalamus. These areas has have previously been shown to be core areas for processing vestibular inputs. The present findings shows that patients with Menière's disease have decreased cerebral activity not only during movement but also when patients are motionless. Furthermore, it support the hypothesis that the medial part of Heschl's gyrus may represent a primary vestibular cortex.

## Data Availability Statement

Requests to share study data and analyses results should be addressed to the corresponding author.

## Ethics Statement

The studies involving human participants were reviewed and approved by Central Region of Denmark Research Ethics Committee approved the study (No: 1-10-72-135-16). The patients/participants provided their written informed consent to participate in this study.

## Author Contributions

TO, J-JM-H, LD, MM, PB, and MP initiated and designed the study. LD included and vestibular tested all trial participants. LD and TL collected PET data. MP was responsible for collection of MRI data. CC collected auditory measurements. AH, PB, and LD conducted data analysis and wrote the main manuscript text. All authors reviewed the manuscript.

### Conflict of Interest

The authors declare that the research was conducted in the absence of any commercial or financial relationships that could be construed as a potential conflict of interest.
